# *Bacillus subtilis* attenuates acetaminophen-induced liver injury and preserves gut–liver homeostasis in mice

**DOI:** 10.1186/s12964-026-03141-2

**Published:** 2026-08-12

**Authors:** Qin Zhuo, Kaiyi Fu, Ziqi Liu, Huilu Zhang

**Affiliations:** 1 Department of Digestive Diseases, Huashan Hospital, Fudan University, 12 Wulumuqi Middle Road, Shanghai, 200040 China; 2 Human Phenome Institute, Fudan University, Shanghai, China; 3 Department of Hepatobiliary and Pancreatic Surgery, the First Affiliated Hospital, Zhejiang University School of Medicine, Hangzhou, China

**Keywords:** Acetaminophen, Acute liver injury, *Bacillus subtilis*, Gut-liver axis, Intestinal barrier, Probiotic

## Abstract

Acetaminophen (APAP) overdose produces an early oxidative insult followed by sterile inflammation and tissue repair, but whether probiotic interventions can influence these distinct phases remains unclear. Here, we evaluated the effects of *Bacillus subtilis* on APAP-induced liver injury in mice. *B. subtilis* pretreatment reduced serum aminotransferases and hepatic necrosis, restored the hepatic GSH/GSSG ratio, and decreased cellular ROS and p-JNK level at 24 h. When *B. subtilis* treatment was initiated after APAP and continued daily, it did not significantly reduce liver injury at 48 h but lowered serum aminotransferases and residual hepatic necrosis at days 5 and 7, consistent with a later recovery-associated benefit. *B. subtilis* pretreatment reduced the frequencies of Ly-6G⁺ neutrophils and total myeloid cells during acute injury, increased the proportion of TIM4⁺ Kupffer cells during recovery, and shifted serum cytokines toward a less inflammatory profile. In parallel, *B. subtilis* preserved colonic morphology and occludin/ZO-1 expression and lowered circulating LPS. Recovery-stage transcriptomic and microbiome analyses associated *B. subtilis* with antioxidant and metabolic programs and altered microbial community structure. Metabolomic analysis identified candidate metabolites elevated in *B. subtilis*-treated mice; among these, histidine and acetyl-L-carnitine were selected for functional testing and provided partial biochemical protection, whereas only live *B. subtilis* reproduced the combined hepatic and intestinal effects. These findings show that viable *B. subtilis* limits acute APAP hepatotoxicity and supports recovery while preserving intestinal barrier integrity.

**Supplementary Information:**

The online version contains supplementary material available at https://doi.org/10.1186/s12964-026-03141-2.

## Introduction

Acetaminophen (APAP; paracetamol) is one of the most widely used over-the-counter antipyretic and analgesic drugs worldwide [1, 2]. Although generally safe at therapeutic doses, overdose can cause severe hepatotoxicity and remains a leading cause of acute liver failure (ALF) [3, 4]. Epidemiological studies indicate that APAP poisoning accounts for a substantial proportion of drug-induced acute liver injury across multiple countries, highlighting an ongoing clinical and public-health burden [5].

The hepatotoxicity of APAP primarily arises from the metabolic formation of N-acetyl-p-benzoquinone imine (NAPQI), a reactive intermediate generated by cytochrome P450 enzymes (CYPs) [6]. During overdose, excessive NAPQI rapidly depletes hepatic glutathione (GSH) and forms protein adducts, triggering redox imbalance, mitochondrial dysfunction, and oxidative stress-associated hepatocyte necrosis [7–9]. Given the central role of oxidative injury in this model, reinforcing antioxidant capacity has been proposed as a therapeutic strategy to limit liver damage.

Currently, N-acetylcysteine (NAC) remains the only clinically approved antidote for APAP-induced hepatotoxicity [10]. NAC replenishes GSH and enhances endogenous antioxidant defense; however, its therapeutic efficacy is limited, particularly in cases of severe overdose, and adverse reactions are not uncommon [11, 12]. These limitations underscore the need for additional interventions that can complement existing treatment and improve outcomes following APAP-induced liver injury.

The gut–liver axis has emerged as an important regulator of hepatic inflammation and metabolic homeostasis, and gut microbes can influence host susceptibility to liver injury [13]. Probiotics and postbiotics have therefore gained interest as modulators of oxidative stress, intestinal barrier integrity, and systemic inflammation [14–19]. However, only a few probiotic strains have been systematically investigated for their therapeutic efficacy, rather than prophylactic efficacy in APAP-induced acute liver injury models [20].

*Bacillus subtilis* is a non-pathogenic, aerobic Gram-positive bacterium with notable resilience and reported immunomodulatory and antioxidant potential [21]. Previous studies suggest that *B. subtilis* supports intestinal homeostasis, competitively inhibits pathogenic colonization, modulates host immune responses, and promotes mucosal recovery [22, 23]. Furthermore, *B. subtilis* supplementation has exhibited beneficial effects in various metabolic and inflammatory diseases, including diet-induced metabolic disorders, diarrhea, constipation, and ulcerative colitis [22–27]. Nonetheless, its impact on oxidative liver injury remains largely unexplored.

Here, we investigated the effects of *B. subtilis* R179 strain in complementary pretreatment and post-treatment models of APAP-induced liver injury and applied integrated transcriptomic, microbiome, and metabolomic analyses. Our findings associate *B. subtilis* treatment with improved liver injury outcomes, phase-dependent hepatic immune and transcriptional changes, preservation of intestinal barrier-associated features, and distinct recovery-stage microbial and metabolic profiles.

## Results

### *B. subtilis* limits acute oxidative hepatotoxicity and supports later recovery

**Fig. 1:**
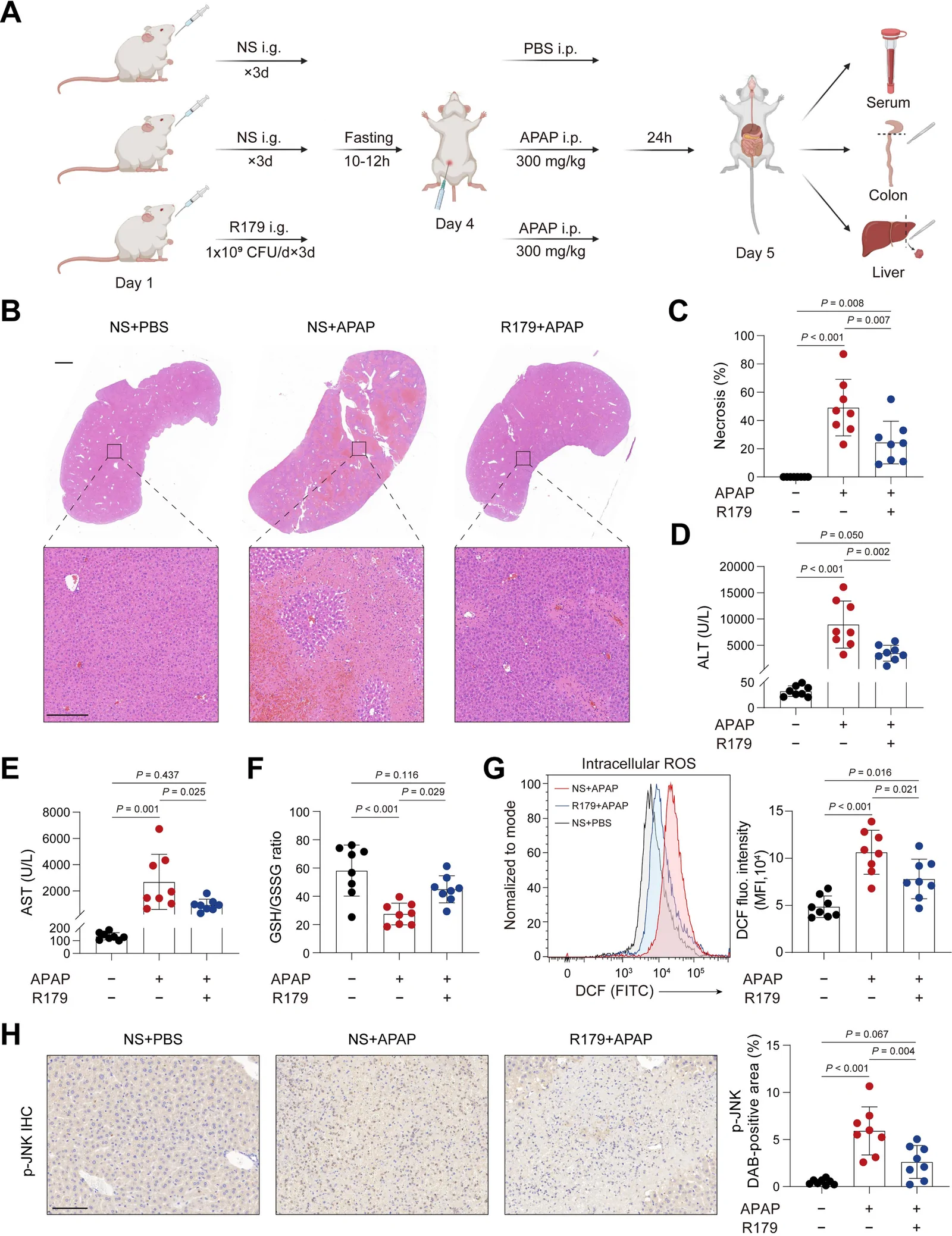
*B. subtilis* protects mice against APAP-induced liver injury. **A** Schematic diagram of the experimental design. Mice were orally administered *B. subtilis* (1 × 10⁹ CFU/day) or normal saline (NS) for 3 consecutive days before APAP challenge. After fasting for 10–12 h, mice were intraperitoneally injected with APAP (300 mg/kg) or PBS and sacrificed 24 h later. i.p., intraperitoneal injection; i.g., intragastric administration; NS, normal saline. **B** Representative H&E staining of liver sections from the indicated groups. Scale bars, 1,000 μm (upper panels) and 100 μm (lower panels). **C** Quantification of hepatic necrotic areas. **D**, **E** Serum levels of ALT (**D**) and AST (**E**) after APAP challenge. **F** Hepatic GSH/GSSG ratios in the indicated groups. **G** Representative flow cytometric analysis of hepatic ROS levels and quantification of DCF fluorescence intensity in the indicated groups. MFI, mean fluorescence intensity. **H** Representative IHC staining of phosphorylated JNK (p-JNK) in liver sections and quantification of p-JNK-positive areas. Scale bars, 100 μm. Data are presented as mean ± SEM. Statistical significance was assessed using one-way ANOVA with Tukey’s multiple-comparisons test for (**C**, **D**, **E**, **F**, **G** and **H**)

To examine the early toxic phase directly, mice received *B. subtilis* R179 or normal saline (NS) for 3 days before APAP and were analyzed 24 h later (Fig. 1A). APAP caused broad centrilobular necrosis and marked elevations in serum ALT and AST, whereas *B. subtilis* pretreatment reduced the necrotic area and lowered both aminotransferases (Fig. 1B–E).

Because APAP injury is largely driven by glutathione depletion and mitochondrial oxidant stress, we next assessed hepatic redox status. APAP decreased the hepatic GSH/GSSG ratio and increased intracellular ROS levels in hepatic cells, whereas *B. subtilis* partially restored the GSH/GSSG ratio and reduced ROS-associated fluorescence (Fig. 1F, G). Phosphorylated JNK immunostaining, an indicator of stress-amplifying JNK activation, was also reduced in *B. subtilis*-treated livers (Fig. 1H).

To determine whether *B. subtilis* remained beneficial when treatment was initiated after APAP-induced injury had begun, we examined an independent cohort in which *B. subtilis* was first administered 1 h after APAP and then continued once daily until sampling (Fig. S1A). At 48 h, ALT, AST, and necrotic area did not differ significantly between the APAP+R179 and APAP+NS groups (ALT, *P* = 0.053; AST, *P* = 0.099; necrotic area, *P* = 0.062; Fig. S1B–C). By days 5 and 7, *B. subtilis* treatment was associated with lower aminotransferase levels and less residual hepatic necrosis (Fig. S1D–G). Together, these findings suggest that *B. subtilis* reduces early injury when administered before APAP and provides a later recovery-associated benefit when treatment is initiated after APAP exposure.

### *B. subtilis* differentially alters hepatic myeloid composition during injury and recovery

Hepatic immune cells, including liver-resident Kupffer cells (KCs) and infiltrating leukocytes, play central roles in sensing tissue damage and coordinating inflammatory responses during liver injury [28]. To determine whether *B. subtilis* altered the hepatic immune landscape after APAP overdose, we isolated hepatic non-parenchymal cells and quantified leukocyte subsets at 24 h, day 5, and day 7.

**Fig. 2:**
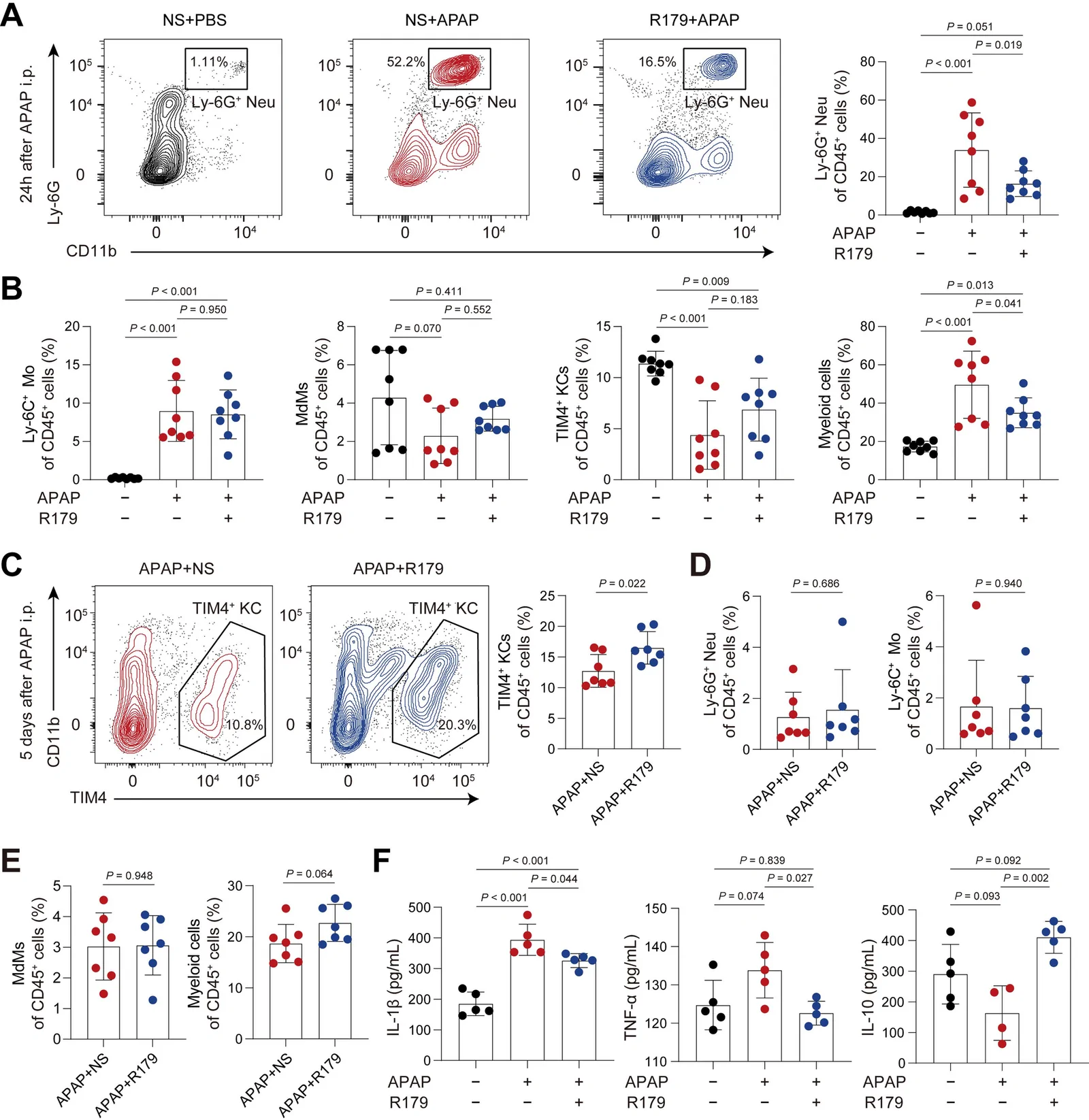
*B. subtilis* modulates hepatic myeloid cell responses during APAP-induced liver injury. **A** Representative flow cytometric plots showing Ly-6G⁺ neutrophils among hepatic CD45⁺ immune cells at 24 h after APAP challenge and quantification of Ly-6G⁺ neutrophil proportions in the indicated groups. **B** Relative proportions of hepatic Ly-6C⁺ monocytes, monocyte-derived macrophages (MdMs), TIM4⁺ Kupffer cells (KCs), and total myeloid cells among CD45⁺ immune cells at 24 h after APAP challenge. (**C**) Representative flow cytometric plots showing TIM4⁺ KCs among hepatic CD45⁺Ly-6G^−^ cells at 5 days after APAP challenge and quantification of TIM4⁺ KC proportions. **D** Relative proportions of Ly-6G⁺ neutrophils and Ly-6C⁺ monocytes among hepatic CD45⁺ immune cells at 5 days after APAP challenge. **E** Relative proportions of MdMs and total myeloid cells among hepatic CD45⁺ immune cells at 5 days after APAP challenge. **F** Serum levels of inflammatory cytokines, including IL-1β, TNF-α, and IL-10, in the indicated groups after APAP challenge. Data are presented as mean ± SEM. Statistical significance was assessed using unpaired two-tailed Student’s *t* test for (**C**, **D** and **E**) and one-way ANOVA with Tukey’s multiple-comparisons test for (**A**, **B** and **F**)

At 24 h, APAP increased the proportion of Ly-6G⁺ neutrophils among CD45⁺ cells, accompanied by an expansion of the overall myeloid compartment. *B. subtilis* attenuated both changes, whereas the frequencies of Ly-6C⁺ monocytes, monocyte-derived macrophages (MdMs), and TIM4⁺ Kupffer cells were not significantly altered (Fig. 2B). By day 5, the difference in neutrophil abundance was no longer evident. Instead, TIM4⁺ Kupffer cells were more abundant in *B. subtilis*-treated livers, whereas neutrophils, monocytes, MdMs, and the overall myeloid compartment were comparable between groups (Fig. 2C–E). We further assessed macrophage phenotypes at day 7 and found that *B. subtilis* increased the proportion of CD206⁺ cells within the macrophage compartment, whereas the frequencies of CD4⁺ T cells, CD8⁺ T cells, and Foxp3⁺ regulatory T cells were unchanged (Fig. S2C–F). In parallel, *B. subtilis* treatment decreased circulating TNF-α and IL-1β and increased IL-10 (Fig. 2F). Together, these findings associate *B. subtilis* treatment with phase-dependent changes in hepatic myeloid composition and a less inflammatory cytokine environment.

### *B. subtilis* is associated with hepatic antioxidant and immune-related transcriptional programs

**Fig. 3:**
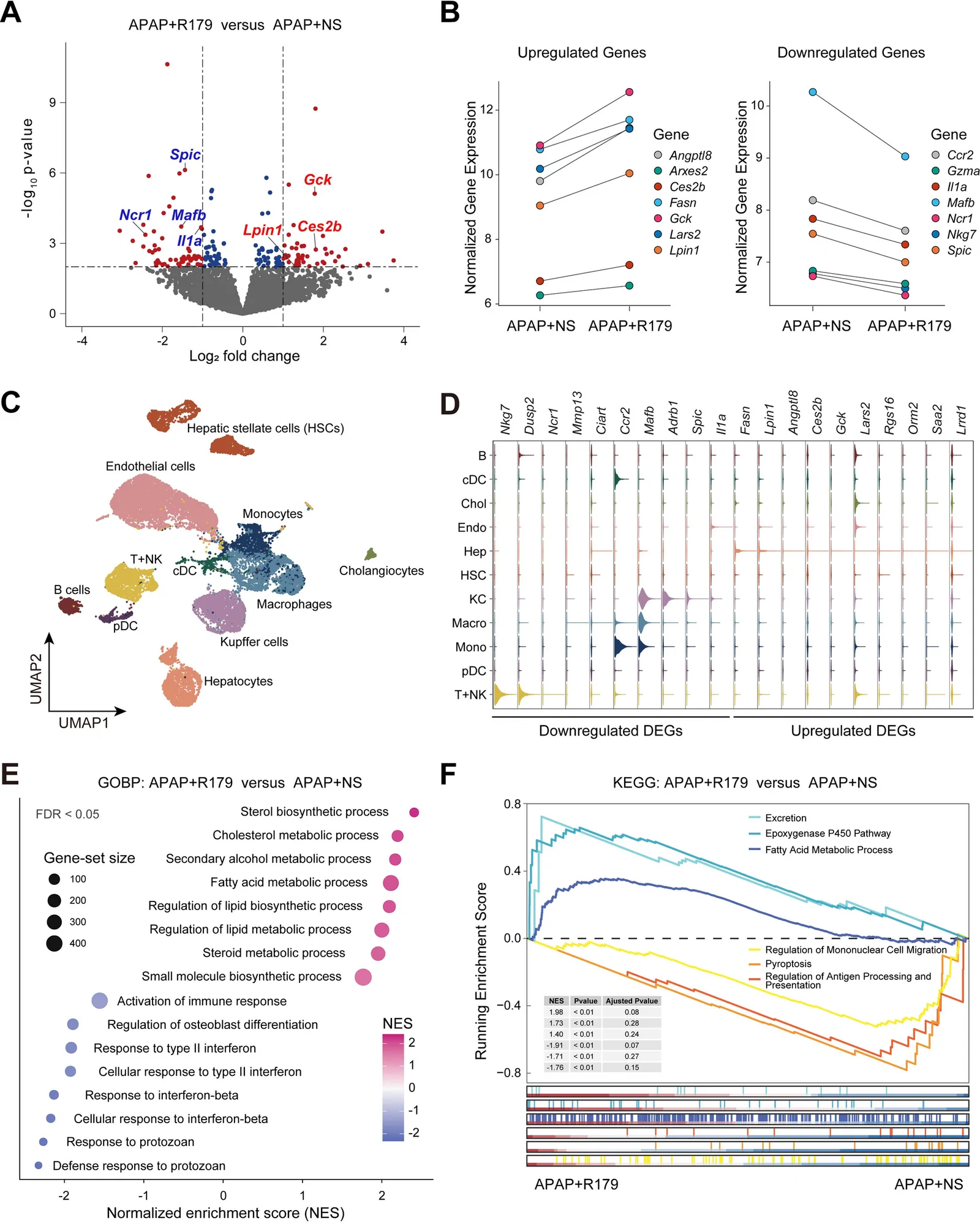
Recovery-stage transcriptomic profiling associates *B. subtilis* with hepatic metabolic and immune-related programs. **A** Volcano plot showing differentially expressed genes (DEGs) between APAP+R179 and APAP+NS groups. **B** Representative upregulated and downregulated genes with the highest log₂ fold changes between APAP+R179 and APAP+NS groups. **C** UMAP visualization of 23,638 single cells from three individual liver samples (GSE166178), identifying 11 major cell populations, including hepatocytes, hepatic stellate cells (HSCs), endothelial cells, cholangiocytes, Kupffer cells, macrophages, monocytes, cDC cells, pDCs, B cells, and T/NK cells. **D** Violin plots showing the expression patterns of representative downregulated DEGs (left) and upregulated DEGs (right) across different liver cell populations. **E** GSEA based on GOBP enrichment pathways between APAP+R179 and APAP+NS groups. **F** GSEA based on KEGG pathway enrichment analysis between APAP+R179 and APAP+NS groups. The curves represent the running enrichment scores. Normalized enrichment score (NES), *P* value, and adjusted *P* value for representative enriched pathways are shown

To characterize the hepatic molecular changes associated with *B. subtilis* treatment during recovery, we performed bulk RNA sequencing of liver tissues collected from the day-7 post-treatment cohort. Using *P* < 0.01 and |log_2_ fold-change| > 1 as thresholds, we identified 180 differentially expressed genes between the APAP+NS and APAP+R179 groups (Fig. 3A). Among them, immune-associated genes, including *Mafb*, *Spic*, *Il1a*, *Ncr1*, *Nkg7*, and *Ccr2*, were expressed at lower levels in *B. subtilis*-treated livers, whereas genes involved in hepatic metabolism, including *Gck*, *Lpin1*, *Ces2b*, and *Lars2*, were expressed at higher levels (Fig. 3B).

To explore the cellular context of these transcriptional differences, we projected the differentially expressed genes onto a public single-cell transcriptomic atlas of APAP-injured mouse liver. Several downregulated genes were preferentially expressed in immune-cell populations, whereas the upregulated metabolic genes were predominantly associated with hepatocytes (Fig. 3C, D). Notably, flow cytometry showed that the proportions of most major hepatic leukocyte populations were comparable between groups at this stage. The transcriptomic differences therefore appear to reflect changes in cellular state and gene expression rather than broad alterations in immune-cell composition.

Gene set enrichment analysis provided additional support for this interpretation. *B. subtilis*-treated livers showed enrichment of gene sets related to lipid and fatty acid metabolism, biosynthesis, cytochrome P450 metabolism, and excretory functions. In contrast, APAP+NS livers were enriched for pathways associated with interferon and immune activation (Fig. 3E, F and Table S3). Gene sets related to ROS responses and Nrf2/antioxidant response element downstream signaling were also enriched in *B. subtilis*-treated livers (Fig. S3A).

To determine whether related transcriptional changes were also detectable during acute injury, we performed quantitative RT-PCR using liver tissues from the independent 24-h pretreatment cohort. *B. subtilis* pretreatment was associated with higher expression of the antioxidant-related genes *Nqo1*, *Gclc*, and *Gclm*, together with lower expression of the myeloid-associated genes *Mafb*, *Ccr2*, and *Spic*, compared with the APAP+NS group (Fig. S3B). Collectively, these findings associate *B. subtilis* treatment with related antioxidant- and myeloid-associated transcriptional changes during APAP-induced liver injury.

### *B. subtilis* preserves the intestinal barrier and reshapes the gut microbiome

**Fig. 4:**
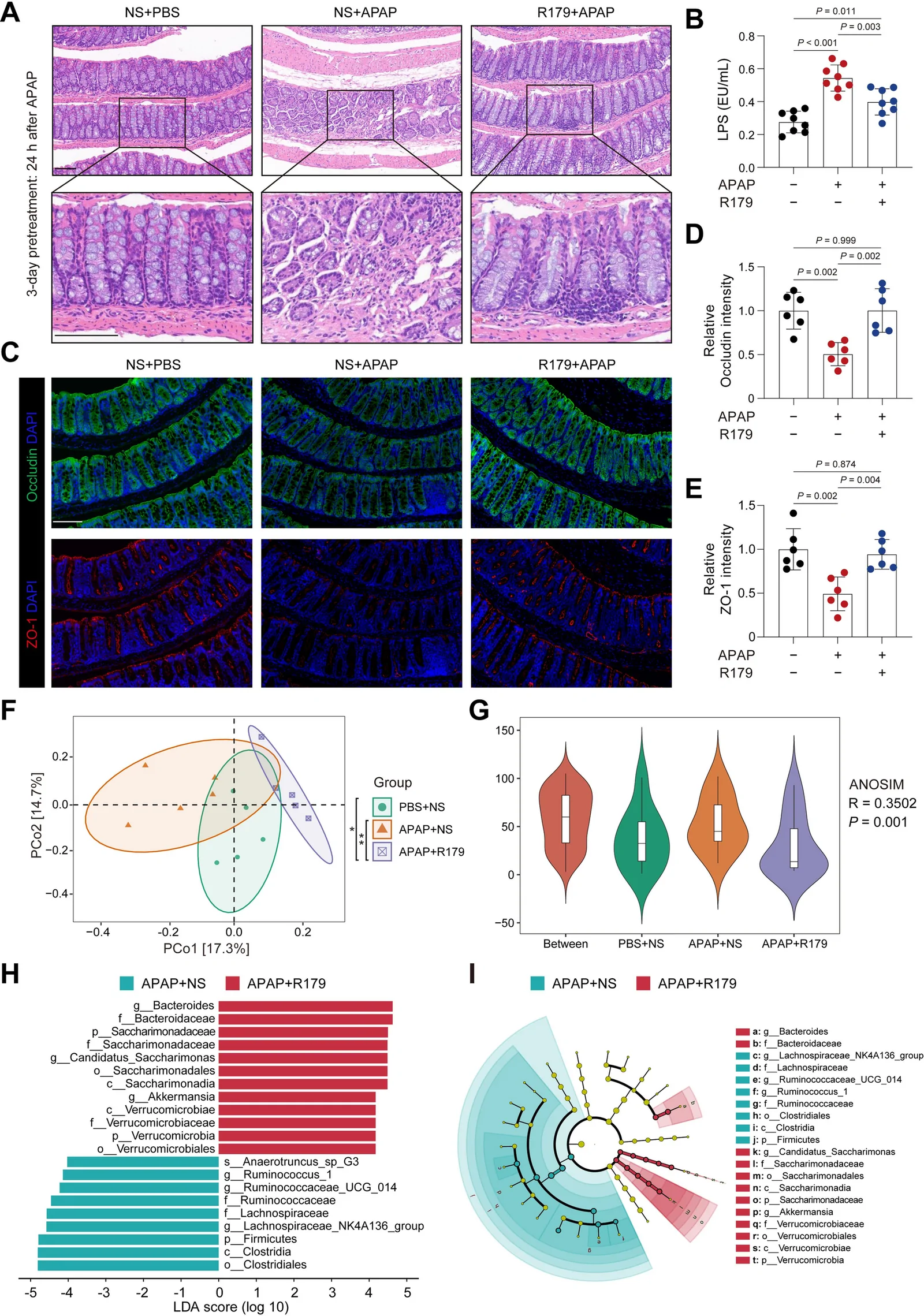
*B. subtilis* preserves intestinal barrier-associated features and alters the recovery-stage microbiota. **A** Representative H&E staining of colon sections from the indicated groups. Scale bars, 100 μm. **B** Serum LPS levels in the indicated groups after APAP challenge. **C** Representative immunofluorescence staining of tight junction proteins in colon sections. Occludin (green), ZO-1 (red), and DAPI (blue). Scale bars, 100 μm. **D**, **E** Quantification of relative fluorescence intensity of occludin (**D**) and ZO-1 (**E**) in colon tissues. **F** Principal coordinates analysis (PCoA) based on Bray–Curtis distance showing differences in gut microbial community composition among the indicated groups. Statistical significance was assessed by PERMANOVA. **G** ANOSIM analysis showing differences in overall microbial community structure among the groups. **H** Linear discriminant analysis effect size (LEfSe) analysis showing differentially abundant bacterial taxa between APAP+R179 and APAP+NS groups. **I** Cladogram showing the taxonomic distribution of differentially enriched taxa. Red and teal nodes indicate taxa enriched in the APAP+R179 and APAP+NS groups, respectively. Statistical significance was assessed using one-way ANOVA with Tukey’s multiple-comparisons test for (**B**, **D** and **E**)

To determine whether the acute hepatoprotective effect of *B. subtilis* was accompanied by preservation of the intestinal barrier, we assessed colonic histology and barrier-associated markers in the 24-hour pretreatment model. APAP disrupted colonic epithelial architecture, increased inflammatory cell infiltration, elevated serum lipopolysaccharide (LPS), and reduced occludin and ZO-1 fluorescence. *B. subtilis* attenuated these changes, as reflected by better-preserved epithelial architecture, reduced apparent inflammatory cell infiltration, lower circulating LPS, and preservation of occludin and ZO-1 signals (Fig. 4A–E). These findings indicate that the acute hepatic benefit of *B. subtilis* coincided with reduced colonic inflammation and preservation of intestinal barrier-associated features.

To examine whether *B. subtilis* treatment was also associated with changes in the gut microbiota during recovery, we profiled cecal microbial communities in the day-7 post-treatment cohort. The number of observed amplicon sequence variants and the Chao1, Shannon, and Simpson diversity indices were comparable among groups (Fig. S4A). Despite the absence of substantial differences in alpha diversity, Bray–Curtis principal-coordinate analysis and ANOSIM indicated differences in overall microbial community structure (Fig. 4F, G). At the phylum level, the APAP+R179 group showed greater relative representation of *Bacteroidetes*, *Saccharibacteria*, and *Verrucomicrobia* (Fig. S4B). At the genus level, heatmap visualization and LEfSe analysis identified enrichment of *Bacteroides*, *Akkermansia*, and *Candidatus Saccharimonas* in *B. subtilis*-treated mice, whereas *Ruminococcaceae_UCG-014*, *Ruminococcus_1*, and *Lachnospiraceae_NK4A136_group*, together with several related *Firmicutes* taxa, were enriched in the APAP+NS group (|LDA score| > 4.0; Fig. 4H, I and Fig. S4C). These findings indicate that *B. subtilis* treatment was associated with a distinct recovery-stage microbial community characterized by enrichment of taxa previously linked to intestinal barrier homeostasis.

Because V3–V4 16 S sequencing lacks sufficient resolution to identify *B. subtilis* at the strain level, we assessed the presence of administered bacteria using complementary analyses. In the 16 S rDNA gene sequencing dataset, the relative abundance of *Bacillales* was higher in the APAP+R179 group than in the APAP+NS group (Fig. S4D). Consistently, quantitative PCR detected substantially greater amounts of fecal *B. subtilis* DNA following R179 administration (Fig. S4E). Together, these complementary findings confirm successful intestinal delivery and fecal detectability of *B. subtilis* during treatment, although the viability and duration of its intestinal residence were not assessed.

To explore the functional potential associated with these compositional changes, we performed Tax4Fun2-based predictive profiling. Compared with the APAP+NS group, the APAP+R179 group showed greater representation of pathways related to glycan, lipid, amino acid, and secondary-metabolite metabolism, but lower representation of membrane transport and genetic-information-processing pathways (Fig. S5A). Similar differences were observed at KEGG level 3, particularly in lipid, amino acid, glycan, energy, and secondary-metabolite metabolism (Fig. S5B). The Sankey diagram further summarized level-2 pathways with higher, lower, or unchanged predicted relative representation in APAP+R179 compared with APAP+NS mice (Fig. S5C). Thus, the *B. subtilis*-associated changes in microbial composition were accompanied by differences in predicted microbial functional potential.

### *B. subtilis* is associated with amino acid-related metabolic changes

**Fig. 5:**
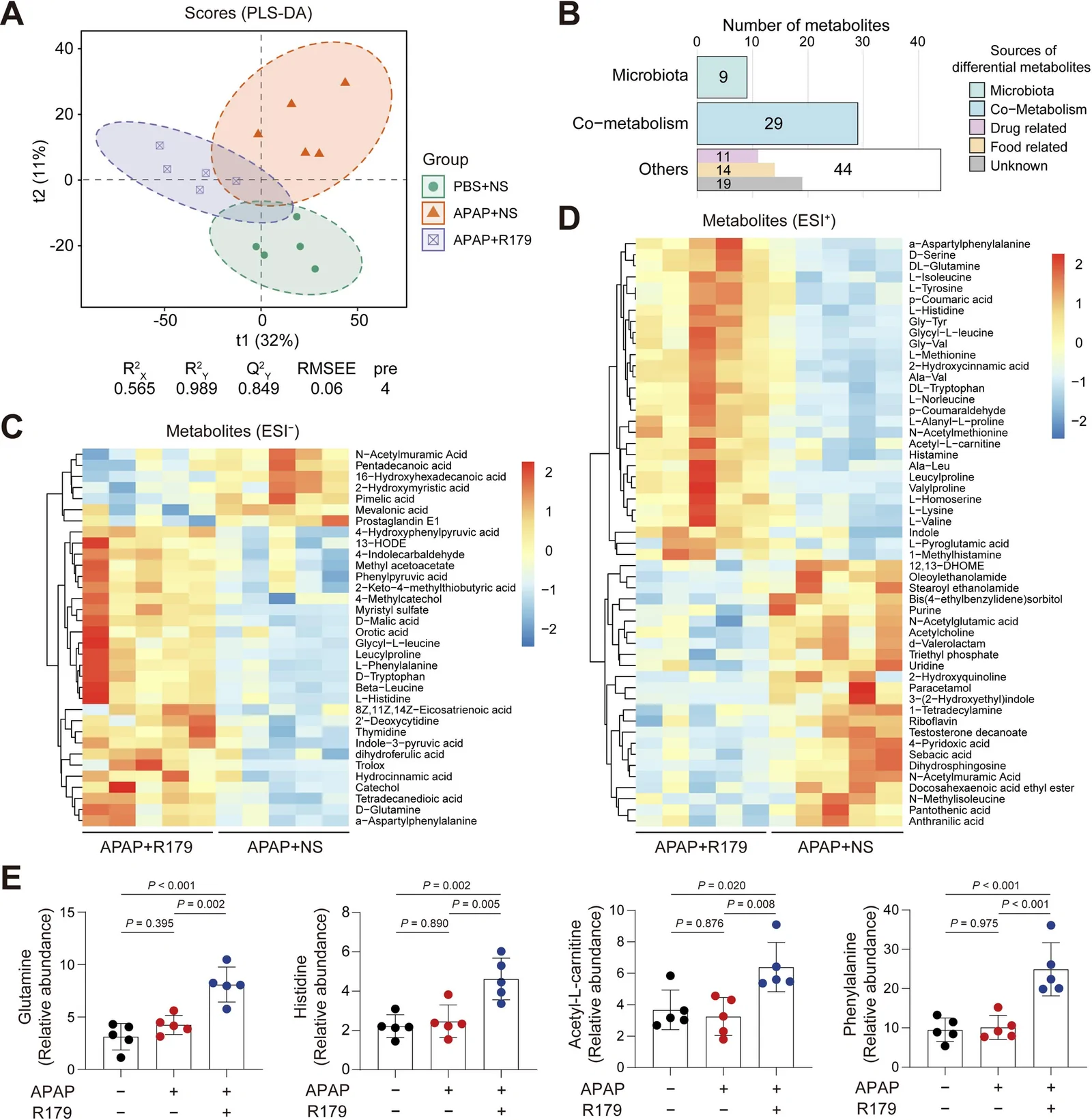
*B. subtilis* is associated with recovery-stage cecal metabolomic changes. **A** Partial least squares discriminant analysis (PLS-DA) score plot of cecal metabolites acquired in ESI⁺ mode from the indicated groups. Ellipses represent Hotelling’s T² 95% confidence intervals. Model performance parameters are shown. **B** Putative source attribution of differential metabolite features, including microbial, host–microbial co-metabolic, and other components. **C**, **D** Heatmaps showing differentially abundant cecal metabolites between APAP+R179 and APAP+NS groups detected in ESI⁻ (**C**) and ESI⁺ (**D**) modes. **E** Relative abundances of representative differential metabolites, including glutamine, histidine, acetyl-L-carnitine, and phenylalanine in the indicated groups. Data are presented as mean ± SEM. Statistical significance was assessed using one-way ANOVA with Tukey’s multiple-comparisons test for (**E**)

To characterize the metabolic changes associated with *B. subtilis* treatment during recovery, we performed untargeted cecal metabolomics in the day-7 post-treatment cohort. PLS-DA showed separation among the three groups in ESI⁺ mode (Fig. 5A), while pairwise PLS-DA and permutation analyses further compared the APAP+NS and APAP+R179 groups in ESI⁻ mode (Fig. S6A–B). Consistent with these overall differences, heatmaps revealed distinct metabolite abundance patterns between the two APAP groups (Fig. 5C–D). Putative source attribution indicated that the differential features included microbial, host–microbial co-metabolic, and other components (Fig. 5B and Table S4).

Pathway enrichment showed that these metabolic differences prominently involved amino acid-related processes, including histidine metabolism, branched-chain amino acid metabolism, and aromatic amino acid biosynthesis and metabolism, together with nucleotide and carbohydrate pathways (Fig. S6C). Exploratory correlation analysis showed that *Bacteroides* and *Akkermansia* were positively associated with several amino acid-related metabolite features, whereas taxa enriched in the APAP+NS group generally showed opposite patterns (Fig. S6D). Among the features enriched in the APAP+R179 group, glutamine, histidine, acetyl-L-carnitine (ALCAR), and phenylalanine were selected as representative candidates and showed higher relative abundance than in the APAP+NS group (Fig. 5E). Together, these results define a *B. subtilis*-associated recovery-stage metabolic profile that parallels changes in the gut microbiota and is characterized by prominent alterations in amino acid-related metabolism.

### Only live *B. subtilis* consistently reproduces the hepatic and intestinal phenotype

**Fig. 6:**
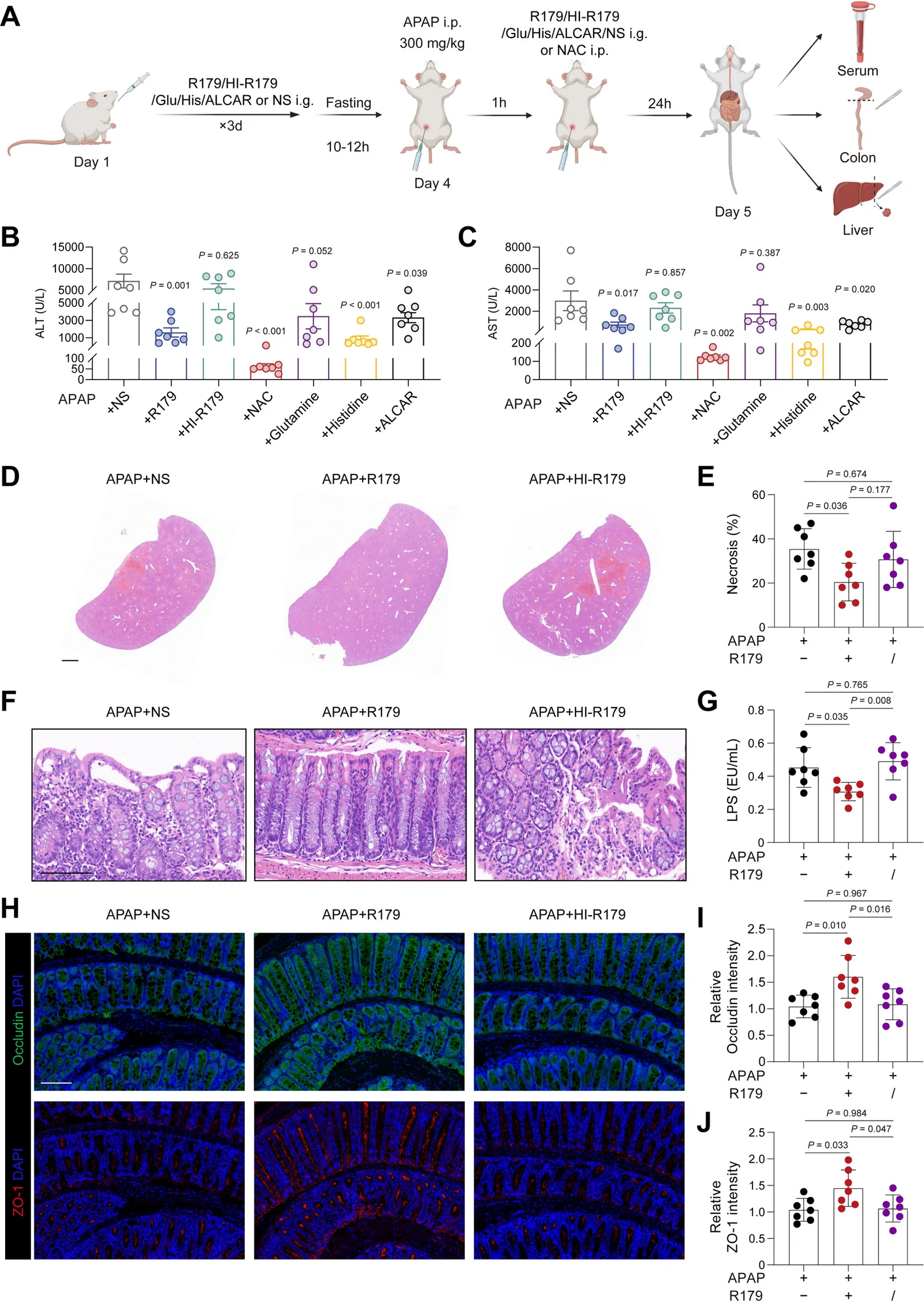
Live *B. subtilis* and selected metabolites contribute to protection against APAP-induced liver injury. **A** Schematic diagram of the experimental design. Mice were orally administered live R179, heat-inactivated R179 (HI-R179), glutamine, histidine, ALCAR, or NS for 3 consecutive days before APAP challenge, followed by an additional dose of the corresponding treatment 1 h after APAP administration. NAC was administered intraperitoneally 1 h after APAP injection as a positive control. i.p., intraperitoneal injection; i.g., intragastric administration. **B**, **C** Serum levels of ALT (**B**) and AST (**C**) in the indicated groups after APAP challenge. **D** Representative H&E staining of liver sections from APAP+NS, APAP+R179, and APAP+HI-R179 groups. Scale bars, 1000 μm. **E** Quantification of hepatic necrotic areas. **F** Representative H&E staining of colon sections from the indicated groups. Scale bars, 100 μm. **G** Serum lipopolysaccharide (LPS) levels in the indicated groups. **H** Representative immunofluorescence staining of tight junction proteins in colon sections. Occludin (green), ZO-1 (red), and DAPI (blue). Scale bars, 100 μm. **I**, **J** Quantification of relative fluorescence intensity of Occludin (**I**) and ZO-1 (**J**) in colon tissues. Data are presented as mean ± SEM. Statistical significance was assessed using one-way ANOVA with Dunnett’s multiple-comparisons test for (**B** and **C**), and one-way ANOVA followed by Tukey’s multiple-comparisons test for (**E**, **G**, **I** and **J**). For (**B** and **C**), each treatment group was compared with the APAP+NS group

To assess the functional relevance of the microbiota-associated metabolic changes identified above and determine whether the effects of *B. subtilis* were retained after heat inactivation, we compared live R179, heat-inactivated R179 (HI-R179), glutamine, histidine, ALCAR, and NAC as a positive control (Fig. 6A). At 24 h after APAP, live R179 reduced serum ALT and AST, whereas HI-R179 produced no significant improvement (Fig. 6B, C). NAC markedly lowered both aminotransferases. Histidine and ALCAR also reduced ALT and AST, whereas glutamine did not consistently improve both measures in our datasets.

**Fig. 7:**
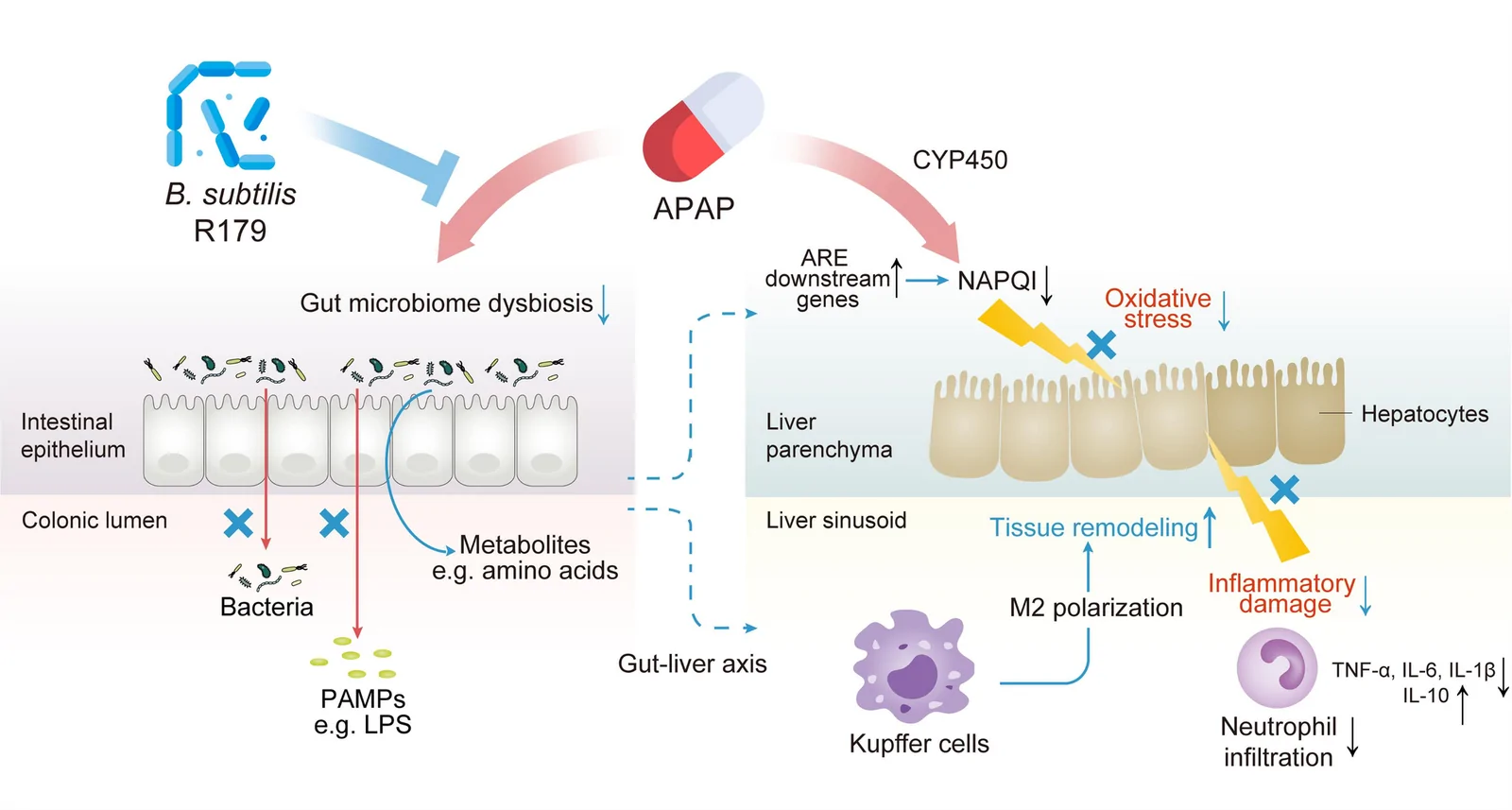
Schematic diagram of the study

At the tissue and intestinal barrier levels, live R179 reduced hepatic necrosis, preserved colonic architecture, decreased inflammatory cell infiltration, lowered circulating LPS, and maintained occludin and ZO-1 signals (Fig. 6D–J). In contrast, HI-R179 did not significantly improve these endpoints relative to the APAP+NS group, and live R179 showed greater effects than HI-R179 on circulating LPS and tight-junction staining (Fig. 6D–J). Thus, the combined hepatic and intestinal phenotype was observed with live R179 but not HI-R179, whereas histidine and ALCAR reproduced a limited biochemical component of the response. Together with the microbiome and metabolome findings, these results support the integrated model summarized in Fig. 7.

## Discussion

It is well known that the gut-liver axis plays a pivotal role in the etiology of many liver diseases [29]. However, among the myriad species of intestinal bacteria, only a few have explicit regulatory effects on liver injury. This study identifies temporally distinct effects of *Bacillus subtilis* R179 in APAP-induced liver injury. *B. subtilis* pretreatment attenuated hepatotoxicity at 24 h, improved hepatic redox balance, reduced hepatic ROS level and p-JNK immunoreactivity, and limited the APAP-induced increase in hepatic neutrophil and myeloid-cell proportions. When *B. subtilis* administration was initiated after APAP and continued daily, serum ALT, AST, and hepatic necrosis did not differ significantly between groups at 48h. In contrast, lower aminotransferase levels and less residual necrosis were observed at days 5 and 7. During recovery, R179 was associated with a higher proportion of Kupffer cells, a higher CD206⁺ macrophage fraction, a less inflammatory circulating cytokine profile, and hepatic transcriptional programs related to metabolism and antioxidant responses. *B. subtilis* also preserved colonic architecture and tight-junction protein signals, reduced circulating LPS, and was associated with distinct recovery-stage microbial and cecal metabolic profiles. Notably, the combined hepatic and intestinal benefits observed with live *B. subtilis* were not retained after heat inactivation. 

The temporal separation between the pretreatment and post-treatment effects is important for interpreting the biological role of *B. subtilis*. APAP bioactivation, glutathione depletion, mitochondrial oxidant stress, and hepatocyte necrosis develop rapidly after overdose, leaving a relatively narrow window for interventions directed at the initiating toxic event [9, 11, 12]. The significant protection observed when *B. subtilis* was administered before APAP indicates that it can reduce susceptibility to the acute insult. By contrast, the absence of a statistically significant post-treatment benefit at 48 h argues against an immediate antidotal effect comparable to that of NAC. The reduction in residual injury at days 5 and 7 instead suggests that *B. subtilis* may influence processes operating during the resolution and repair phases. Because each time point was evaluated in a separate cohort, these results support a later recovery-associated benefit but still do not establish that *B. subtilis* accelerates the rate of injury resolution. 

The hepatic immune response also varied according to the stage of injury. At 24 h, *B. subtilis* reduced the proportions of neutrophils and total myeloid cells, consistent with attenuation of the early inflammatory response. During recovery, neutrophil and monocyte proportions were largely comparable between groups, whereas Kupffer cells were relatively increased at day 5 and the CD206⁺ macrophage fraction was higher at day 7. The accompanying decrease in TNF-α and IL-1β and increase in IL-10 further suggest a shift toward a less inflammatory environment. Together with the day-7 transcriptomic data, these results are more consistent with altered immune-cell states and recovery programs than with broad depletion or expansion of hepatic leukocyte populations. 

A further strength of this study is the integration of hepatic findings with intestinal physiological, microbial, and metabolic measurements. *B. subtilis* preserved colonic architecture and occludin and ZO-1 immunofluorescence and reduced circulating LPS, indicating preservation of intestinal barrier-associated features during acute injury. During recovery, *B. subtilis* was associated with changes in microbial community structure and cecal metabolic profiles rather than a generalized increase in microbial diversity. The enrichment of taxa such as *Akkermansia* may be relevant because members of this genus have been linked to mucus-layer maintenance and epithelial homeostasis, although these effects remain strain- and context-dependent [17, 30]. In parallel, in vivo supplementation with histidine or acetyl-L-carnitine reproduced part of the biochemical benefit by lowering serum aminotransferase levels. However, the 300mg/kg doses used for histidine and ALCAR may exceed the concentrations acheived in vivo through endogenous microbial and host-microbial metabolism. Thus, these experiments support the hepatoprotective potential of these metabolite-associated pathways but do not establish that either metabolite alone mediates the effects of *B. subtilis* at physiologically relevant concentrations. These findings, together with the broader hepatic, intestinal, microbial, and metabolic changes associated with live *B. subtilis*, are more consistent with a multi-component model than with a single-metabolite mechanism.

Several limitations should be considered. First, the high-dose murine APAP model does not fully reproduce the clinical heterogeneity of human acetaminophen toxicity. Second, the present experiments do not determine whether *B. subtilis* acts upstream of APAP bioactivation or primarily through downstream antioxidant and recovery responses. Third, the transcriptomic, microbiome, and metabolomic cohorts were relatively small, and the corresponding analyses remain exploratory. In addition, 16 S sequencing, predicted microbial functions, and untargeted metabolite annotations require higher-resolution and targeted validation. Finally, direct intestinal permeability measurements, microbiota depletion or transfer, strain-specific tracking, metabolite tracing, and host or bacterial loss-of-function studies will be required to establish causal gut-to-liver mediation.

In conclusion, *B. subtilis* reduced susceptibility to acute APAP hepatotoxicity when administered before injury and was associated with lower residual liver injury when treatment was initiated after APAP. Its effects were accompanied by improved redox homeostasis, phase-dependent changes in hepatic myeloid populations, preservation of intestinal barrier-associated features, and coordinated recovery-stage microbial and metabolic changes. These findings support further evaluation of live *B. subtilis* as a potential adjunctive intervention and identify hepatic antioxidant responses, intestinal barrier regulation, and amino acid-related metabolism as priority targets for mechanistic investigation.

## Methods

### Animals and ethics

Male and female BALB/c mice (5 weeks old; 20 ± 1 g) were obtained from Jiangsu Jicui Yaokang Biological Technology Co., Ltd. (Nanjing, China) and maintained in sterile individually ventilated cages at 25 °C and 55% relative humidity under a 12 h light/dark cycle with standard chow and water available ad libitum. Mice were allocated to the indicated experimental groups and fasted for 10–12 h before APAP administration. Animal procedures were approved by the Ethics Committee of the Department of Laboratory Animal Science, Fudan University (approval no. 2021JS-Huashan-392) and were performed in accordance with the approved protocol.

### Modeling of APAP-induced liver injury in mice

*Bacillus subtilis* R179 was provided by Beijing Hanmi Pharmaceutical Co., Ltd. (Beijing, China). R179 was administered by oral gavage at 1 × 10⁹ colony-forming units (CFU) per mouse per day in normal saline (NS); control mice received the same volume of NS. APAP (Absin, Shanghai, China) was freshly dissolved in warm phosphate-buffered saline (PBS) and administered intraperitoneally at 300 mg/kg; control mice received PBS.

For the acute pretreatment model, mice received NS or R179 once daily for 3 consecutive days. After overnight fasting, mice received PBS or APAP on day 4 and were sampled 24 h later. For the post-treatment time-course experiment, mice received APAP and then began NS or R179 gavage 1 h later; treatment was continued once daily until sampling at 48 h, day 5, or day 7. For immune kinetic analyses, liver non-parenchymal cells were collected at 24 h, day 5 and day 7 after APAP. For recovery-stage bulk RNA sequencing, 16 S rDNA sequencing, and untargeted metabolomics, samples were derived from the separate 7-day post-treatment cohort described above.

### Heat-inactivated R179, metabolite supplementation, and NAC treatment

To obtain heat-inactivated R179, freshly prepared R179 suspensions were adjusted to the desired concentration based on viable bacterial counts and subsequently inactivated by autoclaving at 121 °C under 103 kPa pressure for 20 min. After cooling to room temperature, the bacterial suspension was thoroughly mixed and administered to mice at a dose equivalent to 1 × 10⁹ CFU before inactivation, using the same gavage volume and treatment schedule as those used for live R179. Complete loss of bacterial viability was confirmed by plating an aliquot of the autoclaved suspension on LB agar and incubating at 37 °C for 48 h. No bacterial colonies were detected after incubation.

To evaluate whether candidate metabolites contributed to the protective effects of R179, separate groups of mice received glutamine, histidine, or acetyl-L-carnitine hydrochloride (ALCAR) by oral gavage. Glutamine and histidine were purchased from BBI Life Sciences (China; catalog nos. A600224 and A604351, respectively), and acetyl-L-carnitine hydrochloride was purchased from Macklin Biochemical (China; catalog no. A834960). Each metabolite was administered at 300 mg/kg body weight once daily for 3 consecutive days before APAP challenge, followed by an additional dose administered 1 h after APAP injection. N-acetyl-L-cysteine (NAC; Beyotime Biotechnology, China; catalog no. S0077) was administered intraperitoneally at 300 mg/kg 1 h after APAP injection. Control mice received the corresponding vehicle using the same route and dosing schedule.

### Serum biochemical and cytokine measurements

Serum ALT and AST activities were measured using the corresponding Siemens reagent kits on an automated clinical chemistry analyzer (ADVIA Chemistry XPT System, Siemens Healthcare Diagnostics Inc.) according to the manufacturer’s instructions. Serum IL-1β, TNF-α, and IL-10 concentrations were determined using mouse-specific enzyme-linked immunosorbent assay kits (R&D Systems, Minneapolis, MN, USA) according to the manufacturer’s protocols.

### Measurement of serum lipopolysaccharide

Serum lipopolysaccharide (LPS) levels were determined using a chromogenic Limulus amebocyte lysate endotoxin assay kit (Beyotime Biotechnology, China; C0276) according to the manufacturer’s instructions. Serum samples were incubated with LAL reagent at 37 °C, followed by addition of the chromogenic substrate. Absorbance was measured at 545 nm using a microplate reader. Endotoxin concentrations were calculated from the standard curve and expressed as endotoxin units per milliliter (EU/mL).

### Histology

Liver and colon tissues were fixed in 4% neutral-buffered formalin, embedded in paraffin, sectioned at 5 μm, and stained with hematoxylin and eosin (H&E). Hepatic necrosis was quantified as the percentage of necrotic area relative to the total liver tissue area using QuPath software (version 0.7.0). Multiple non-overlapping fields were analyzed for each mouse, and the mean value was used as one biological replicate.

### Determination of hepatic GSH/GSSG ratio

Hepatic reduced glutathione (GSH) and oxidized glutathione (GSSG) were measured using a Total Glutathione/Oxidized Glutathione Assay Kit (Nanjing Jiancheng Bioengineering Institute, China) according to the manufacturer’s instructions. Liver tissues were homogenized in ice-cold extraction buffer and centrifuged to collect the supernatants. Total glutathione and GSSG were determined colorimetrically, and the GSH/GSSG ratio was calculated to assess hepatic redox status.

### Measurement of hepatic cellular ROS

Intracellular reactive oxygen species (ROS)-associated fluorescence was measured using a ROS assay kit (Nanjing Jiancheng Bioengineering Institute, China; E004-1) based on the oxidation-sensitive probe 2’,7’-dichlorodihydrofluorescein diacetate (DCFH-DA). Briefly, liver tissues were collected from mice in the indicated treatment groups and processed into single-cell suspensions. Cells were incubated with the DCFH-DA working solution at 37 °C in the dark for 30 min. After incubation, the cells were washed with PBS to remove excess probe and stained with an anti-mouse CD45 antibody. Following exclusion of debris and doublets, hepatic CD45^−^ cells were gated, and 2’,7’-dichlorofluorescein (DCF) fluorescence intensity within this population was quantified by flow cytometry.

### Isolation of hepatic non-parenchymal cells

Mice were euthanized, and the abdominal surface was disinfected with 70% ethanol. The abdominal cavity was opened, and the liver was gently exposed. After removal of the gallbladder and dissection of the surrounding ligaments, the liver was excised and immediately placed in ice-cold RPMI 1640 medium. Liver tissues were minced into approximately 1–2 mm³ pieces and further mechanically disrupted using the plunger of a syringe. The tissue fragments were transferred to a 50 mL centrifuge tube containing 10 mL of prewarmed digestion medium consisting of RPMI 1640 supplemented with collagenase I, hyaluronidase and DNase I. The tissues were incubated at 37 °C for 30 min, with gentle agitation every 10 min. The resulting cell suspension was filtered through a 70 μm cell strainer and was centrifuged at 50 × g for 3 min to sediment hepatocytes and large tissue debris. The supernatant was carefully collected and centrifuged at 400 × g for 5 min to obtain hepatic non-parenchymal cells. The cell pellet was resuspended in ACK red blood cell lysis buffer and incubated for 30–60 s, terminated by adding 20 mL RPMI 1640. The cells were then resuspended in 10 mL ice-cold RPMI 1640, filtered again through a 70 μm cell strainer, and centrifuged at 400 × g for 5 min. The final hepatic non-parenchymal cell pellet was resuspended in PBS for flow cytometric staining.

### Flow cytometry

Isolated hepatic non-parenchymal cells were resuspended in 200 µL PBS. Fc receptors were blocked with an anti-mouse CD16/CD32 antibody for 10 min at 4 °C. Aliquots of the cell suspension were used to prepare unstained and single-stained compensation controls. The remaining cells were incubated with the indicated fluorophore-conjugated antibody mixture for 30 min at 4 °C in the dark. For Foxp3 staining, surface-stained cells were fixed and permeabilized using a transcription-factor staining buffer set according to the manufacturer’s protocol, followed by intracellular staining with anti-mouse Foxp3 antibody. After staining, the cells were washed with PBS and resuspended for flow cytometric acquisition. Data were acquired using a CytoFLEX (Beckman Coulter, Miami, FL, USA) flow cytometer and analyzed using FlowJo software version 10.8.1 (BD Biosciences). The flow cytometry gating strategy was depicted in Fig. S2A–B. Uniform Manifold Approximation and Projection (UMAP) analysis was performed using the FlowJo plugin UMAP version 3.1 (with 15 neighbors, metric = Euclidean, and minimum distance = 0.5 as default parameters). Details of all antibodies used are provided in Table S1.

### Immunofluorescence staining

Paraffin-embedded colon sections were used for immunofluorescence staining of the tight-junction proteins occludin and ZO-1. The sections were deparaffinized, rehydrated, and subjected to heat-induced antigen retrieval in 0.01 M citrate buffer (pH 6.0) at 100 °C for 10 min. After cooling, the sections were blocked with 5% bovine serum albumin for 30 min at room temperature and incubated overnight at 4 °C with primary antibodies against Occludin (Proteintech, 27260-1-AP, 1:200) or ZO-1 (Proteintech, 66452-1-Ig, 1:200). After washing with PBS, the sections were incubated with the corresponding fluorophore-conjugated secondary antibodies for 1 h at room temperature in the dark. Nuclei were counterstained with DAPI, and the sections were mounted with antifade mounting medium. Images were acquired using an Olympus FV3000i confocal microscope with FV31S-SW software under identical imaging settings. Occludin and ZO-1 fluorescence intensities were quantified using ImageJ software, and the average value from multiple non-overlapping fields was calculated for each mouse.

### Immunohistochemical staining

Paraffin-embedded liver sections were baked at 60 °C for 2 h, deparaffinized in xylene, and rehydrated through a descending ethanol series. Endogenous peroxidase activity was quenched by incubation with 3% hydrogen peroxide for 30 min at room temperature. Heat-induced antigen retrieval was performed by microwave heating in citrate buffer, after which the sections were allowed to cool to room temperature and washed with PBS.

Nonspecific binding was blocked with 5% bovine serum albumin for 30 min at room temperature. The sections were subsequently incubated overnight at 4 °C with an antibody against phospho-JNK (Tyr185; Proteintech, catalog no. 80024-1-RR). After washing, the sections were incubated with the corresponding horseradish peroxidase-conjugated secondary antibody for 30–60 min at 37 °C. Immunoreactivity was visualized using 3,3’-diaminobenzidine substrate, and color development was terminated by rinsing the sections under running water. The nuclei were counterstained with hematoxylin. Finally, the sections were dehydrated through a graded ethanol series, cleared in xylene, and mounted with neutral resin. Hepatic phospho-JNK immunoreactivity was quantified as the percentage of DAB-positive area within the analyzed liver tissue. Multiple non-overlapping fields were analyzed for each mouse, and the mean value was used as one biological replicate.

### RNA extraction, reverse transcription, and quantitative PCR

Total RNA was extracted from the liver using TRIzol (Thermo Fisher Scientific), according to the manufacturer’s protocol. RNA quantity and quality were analyzed using a NanoDrop 2000 (Thermo Fisher Scientific). Reverse transcription was performed using PrimeScript RT Reagent Kit (Takara, Shiga, Japan) according to the provided instructions. Real-time quantitative RT-PCR was performed using a CFX Connect real-time system (Bio-Rad, Hercules, CA, USA) and RNA levels were quantified using the SYBR Green Master Mix (Vazyme, Nanjing, China). β-actin RNA levels were used for data normalization. Primer sequences are listed in Table S2.

### Quantification of *B. subtilis* DNA

Microbial genomic DNA was extracted from individual fecal pellets using the MolPure Stool DNA Kit (Yeasen Biotechnology, China) according to the manufacturer’s instructions. *B. subtilis*-specific primers were forward 5’-GGCCTATTGAACACCCTGATTTA-3’ and reverse 5’-CGGATGCGGCCTTCTTTTTC-3’. Quantitative PCR was performed with SYBR Green Master Mix on a real-time PCR system. Relative *B. subtilis* DNA abundance was normalized to total bacterial 16 S rDNA gene abundance and calculated using the 2^⁻ΔCt^ method.

### Bulk RNA-sequencing

Total RNA from each liver sample (*n* = 3 per group) was quantified and quality-assessed using an Agilent 2100 Bioanalyzer. One microgram of total RNA with an RNA integrity number greater than 6.5 was used for library preparation. Next-generation sequencing library preparations were constructed according to the manufacturer’s protocol. The poly(A) mRNA isolation was performed using a Poly(A) mRNA Magnetic Isolation Module or rRNA removal Kit. The mRNA fragmentation and priming were performed using First Strand Synthesis Reaction Buffer and Random Primers. First-strand cDNA was synthesized using ProtoScript II Reverse Transcriptase and the second-strand cDNA was synthesized using Second Strand Synthesis Enzyme Mix. The purified double-stranded cDNA by beads was then treated with End Prep Enzyme Mix to repair both ends and add a dA-tailing in one reaction, followed by a T-A ligation to add adaptors to both ends. Size selection of adaptor-ligated DNA was then performed using beads, and fragments of ~ 420 bp (with the approximate insert size of 300 bp) were recovered. Each liver sample was then amplified by PCR for 13 cycles using P5 and P7 primers, with both primers carrying sequences that can anneal with flow cell to perform bridge PCR and P7 primer carrying a six-base index allowing for multiplexing. The PCR products were cleaned up using beads, validated using a Qsep100 (Bioptic, Taiwan, China), and quantified by Qubit 3.0 Fluorometer (Invitrogen, Carlsbad, CA, USA). Then libraries with different indices were multiplexed and loaded on an Illumina HiSeq instrument according to the manufacturer’s instructions (Illumina, San Diego, CA, USA). Sequencing was carried out using a 2 × 150 bp paired-end (PE) configuration; image analysis and base calling were conducted by the HiSeq Control Software (HCS) + OLB + GAPipeline-1.6 (Illumina) on the HiSeq instrument.

Raw fastq sequencing reads were aligned against the reference genome sequence (GRCm38/mm10) using the HISAT2 (v2.0.1), discarding all non-uniquely aligned reads. Differentially expressed genes (DEGs) were identified by the DESeq2 package in R (version 4.3.1). Ranked genes were aligned against the KEGG (Kyoto Encyclopedia of Genes and Genomes), Gene Ontology Biological Process (GOBP), or Hallmark database for gene set enrichment analysis (GSEA) by R package clusterProfiler (version 4.4.4). Gene-list enrichment of KEGG was done by KOBAS (http://kobas.cbi.pku.edu.cn/).

### Public single-cell RNA-seq mapping

The scRNA-seq dataset of APAP-induced liver injury mice, namely, GSE166178 [31] (sample GSM5065210-GSM5065212) was downloaded from the Gene Expression Omnibus (GEO) database. The R package Seurat (version 4.2.0) was used for downstream analysis and visualization. As a quality control criterion, we included the cells containing at least 500 genes, 1000 counts, and less than 30% mitochondrial transcript content. The filtered expression matrix was processed using SCTransform. Principal component analysis (PCA) was done using the Seurat RunPCA function to calculate the top 40 principal components (PCs). Visualization and clustering were done in Seurat following the official vignette of RunUMAP, DimPlot, and VlnPlot functions using default parameters.

### 16 S rDNA gene sequencing and microbiome analysis

The contents of the cecum were homogenized in PBS and immediately frozen (-80 °C) and stored until further detection and analysis. 16 S rDNA sequencing was performed by Illumina MiSeq at GENEWIZ (Shanghai, China). In short, 20–30 ng DNA was used to generate amplicons. V3 and V4 hypervariable regions of prokaryotic 16 S rDNA were selected for generating amplicons and following taxonomy analysis. The QIIME data analysis package was used for 16 S rDNA data analysis, and the USEARCH system was used to remove chimeric sequences. Sequences were grouped into amplicon sequence variants (ASVs) against the Silva database.

The ASV abundance at each taxonomic rank, the alpha diversity (Chao1, Shannon, Simpson indices) and the beta diversity distance (based on principal coordinate analysis, PCoA) were calculated using R package microeco (version 0.11.0). The permutational multivariate analysis of variance (PERMANOVA) and the analysis of similarities (ANOSIM) were used to test the significance of distances among groups with the R package vegan (version 2.6-2). Linear discriminant analysis effect size (LEfSe) analysis was utilized to identify differentially abundant taxa between the groups using the software LEFSE. The linear discriminant analysis (LDA) score threshold was set to 4.0. To putatively predict microbial functions from 16 S rDNA gene sequencing data, R package Tax4Fun2 (version 1.1.5) and the KEGG database were used. Extended error bar plots of KEGG orthologues were generated using the Statistical Analysis of Metagenomic Profiles (STAMP) software using Welch’s *t*-test.

### Metabolomics analysis

Untargeted metabolomics of cecal contents were performed using liquid chromatography-mass spectrometry (LC-MS). The frozen-thawed samples (*n* = 5 for each group) were extracted with 80% methanol (Merck, Darmstadt, Germany) and ground to a fine powder using a grinding mill at 65 Hz for 90 s, then sonicated at 40 kHz for 30 min and left for 1 h at -40 °C. After 15 min of centrifugation at 12,000 rpm at 4 °C, 200 µL supernatant of each sample was transferred to vials to perform LC-MS analysis. Cecal metabolomic profiling was performed using a Waters UPLC system coupled to a Q Exactive Orbitrap mass spectrometer and equipped with an ACQUITY UPLC HSS T3 column (1.8 μm, 2.1 × 100 mm). Mobile phase A was water with 0.05% formic acid, and mobile phase B was acetonitrile. The automatic injector temperature was 4 °C, the injection volume was 5 µL, the column temperature was 40 °C, and the flow rate was 0.3 mL/min. Centroid data were collected from 70 to 1050 m/z.

The pretreatments on acquired MS data were performed using the XCMS software (Scripps Research Institute, USA). Detailed methods for LC-MS raw data file processing, calculation of the exact molecular mass data (m/z), online KEGG and HMDB (http://www.hmdb.ca/spectra/ms/search) database annotation, and quality control have been described previously [32]. Tail area-based false discovery rate (FDR) correction (q-value) was applied to strictly control for false positives. Metabolites were considered as biomarkers if they fulfilled the following standards: (1) q < 0.05, P value using two-sided Mann-Whitney U test corrected by FDR, and (2) variable importance for the projection (VIP) > 1 using partial least squares discriminant analysis (PLS-DA). MetOrigin software was used to perform metabolite functional enrichment analysis and metabolite traceability analysis. Integrative analysis of the microbiome and metabolome was conducted using Spearman’s correlation test.

### Statistical analysis

Statistical analyses were performed using GraphPad Prism (v9.5.0), R (v4.3.1) and the software indicated for individual omics workflows. Data are shown as mean ± SEM, with each point representing an independent biological replicate. Two-group comparisons used two-tailed unpaired Student’s *t* tests when the assumptions of normality and homogeneity of variance were met. For comparisons among three or more groups, one-way analysis of variance (ANOVA) followed by Tukey’s or Dunnett’s multiple-comparisons test was used when the assumptions of normality and homogeneity of variance were met. Exact *P* values are displayed in the figures whenever available. *P* < 0.05 was considered statistically significant. Omics-specific testing and multiplicity corrections are described above.

## Supplementary Information

Supplementary Material 1.

## Data Availability

The original contributions presented in the study are included in the article/Supplementary Material, further inquiries can be directed to the corresponding authors.

## References

[1] Freo U, Ruocco C, Valerio A, Scagnol I, Nisoli E. Paracetamol: a review of guideline recommendations. J Clin Med. 2021;10. 10.3390/jcm10153420 PMC8347233 34362203

[2] Okumura Y, Kimura T, Inokuchi S, Okamoto M, Takano T, Miyake M, et al. Real-World Hepatic Safety of Oral Acetaminophen Versus Loxoprofen/Celecoxib: Using a Nationwide Electronic Medical Record Database. J Clin Pharmacol. 2023;63:798–806. 10.1002/jcph.2216 36841952

[3] Bunchorntavakul C, Reddy KR. Acetaminophen (APAP or N-Acetyl-p-Aminophenol) and Acute Liver Failure. Clin Liver Dis. 2018;22:325–46. 10.1016/j.cld.2018.01.007 29605069

[4] Yoon E, Babar A, Choudhary M, Kutner M, Pyrsopoulos N. Acetaminophen-Induced Hepatotoxicity: a Comprehensive Update. J Clin Transl Hepatol. 2016;4:131–42. 10.14218/JCTH.2015.00052 PMC4913076 27350943

[5] Lee WM. Acetaminophen (APAP) hepatotoxicity-Isn’t it time for APAP to go away? J Hepatol. 2017;67:1324–31. 10.1016/j.jhep.2017.07.005 PMC5696016 28734939

[6] Athersuch TJ, Antoine DJ, Boobis AR, Coen M, Daly AK, Possamai L, et al. Paracetamol metabolism, hepatotoxicity, biomarkers and therapeutic interventions: a perspective. Toxicol Res (Camb). 2018;7:347–57. 10.1039/c7tx00340d PMC6062253 30090586

[7] McGill MR, Sharpe MR, Williams CD, Taha M, Curry SC, Jaeschke H. The mechanism underlying acetaminophen-induced hepatotoxicity in humans and mice involves mitochondrial damage and nuclear DNA fragmentation. J Clin Invest. 2012;122:1574–83. 10.1172/JCI59755 PMC3314460 22378043

[8] More SS, Nugent J, Vartak AP, Nye SM, Vince R. Hepatoprotective Effect of psi-Glutathione in a Murine Model of Acetaminophen-Induced Liver Toxicity. Chem Res Toxicol. 2017;30:777–84. 10.1021/acs.chemrestox.6b00291 28165728

[9] Yan M, Huo Y, Yin S, Hu H. Mechanisms of acetaminophen-induced liver injury and its implications for therapeutic interventions. Redox Biol. 2018;17:274–83. 10.1016/j.redox.2018.04.019 PMC6006912 29753208

[10] European Association for the Study of. the L. EASL Clinical Practice Guidelines: Drug-induced liver injury. J Hepatol. 2019;70:1222–61. 10.1016/j.jhep.2019.02.014 30926241

[11] Du K, Ramachandran A, Jaeschke H. Oxidative stress during acetaminophen hepatotoxicity: Sources, pathophysiological role and therapeutic potential. Redox Biol. 2016;10:148–56. 10.1016/j.redox.2016.10.001 PMC5065645 27744120

[12] Jaeschke H, Akakpo JY, Umbaugh DS, Ramachandran A. Novel Therapeutic Approaches Against Acetaminophen-induced Liver Injury and Acute Liver Failure. Toxicol Sci. 2020;174:159–67. 10.1093/toxsci/kfaa002 PMC7098369 31926003

[13] Dewanjee S, Dua TK, Paul P, Dey A, Vallamkondu J, Samanta S, et al. Probiotics: Evolving as a Potential Therapeutic Option against Acetaminophen-Induced Hepatotoxicity. Biomedicines. 2022;10. 10.3390/biomedicines10071498 PMC9312935 35884803

[14] Ahmed HM, Shehata HH, El-Saeed GSM, Gabal HHA, El-Daly SM. Ameliorative effect of Lactobacillus rhamnosus GG on acetaminophen-induced hepatotoxicity via PKC/Nrf2/PGC-1alpha pathway. J Genet Eng Biotechnol. 2022;20:142. 10.1186/s43141-022-00422-4 PMC9537380 36201094

[15] Jiang H, Yan R, Wang K, Wang Q, Chen X, Chen L, et al. Lactobacillus reuteri DSM 17938 alleviates d-galactosamine-induced liver failure in rats. Biomed Pharmacother. 2021;133:111000. 10.1016/j.biopha.2020.111000 33202285

[16] Lv L, Yao C, Yan R, Jiang H, Wang Q, Wang K, et al. Lactobacillus acidophilus LA14 Alleviates Liver Injury. mSystems. 2021;6:e0038421. 10.1128/mSystems.00384-21 PMC8269229 34128694

[17] Xia J, Lv L, Liu B, Wang S, Zhang S, Wu Z, et al. Akkermansia muciniphila Ameliorates Acetaminophen-Induced Liver Injury by Regulating Gut Microbial Composition and Metabolism. Microbiol Spectr. 2022;10:e0159621. 10.1128/spectrum.01596-21 PMC8809353 35107323

[18] Yan R, Wang K, Wang Q, Jiang H, Lu Y, Chen X, et al. Probiotic Lactobacillus casei Shirota prevents acute liver injury by reshaping the gut microbiota to alleviate excessive inflammation and metabolic disorders. Microb Biotechnol. 2022;15:247–61. 10.1111/1751-7915.13750 PMC8719798 33492728

[19] Yang L, Bian X, Wu W, Lv L, Li Y, Ye J, et al. Protective effect of Lactobacillus salivarius Li01 on thioacetamide-induced acute liver injury and hyperammonaemia. Microb Biotechnol. 2020;13:1860–76. 10.1111/1751-7915.13629 PMC7533332 32652882

[20] Wu Y, Wang B, Tang L, Zhou Y, Wang Q, Gong L, et al. Probiotic Bacillus Alleviates Oxidative Stress-Induced Liver Injury by Modulating Gut-Liver Axis in a Rat Model. Antioxid (Basel). 2022;11. 10.3390/antiox11020291 PMC8868294 35204173

[21] Suva M, Sureja V, Kheni D. Novel insight on probiotic Bacillus subtilis: Mechanism of action and clinical applications. J Curr Res Sci Med. 2016;2:65–72.

[22] Kim B, Kwon J, Kim MS, Park H, Ji Y, Holzapfel W, et al. Protective effects of Bacillus probiotics against high-fat diet-induced metabolic disorders in mice. PLoS ONE. 2018;13:e0210120. 10.1371/journal.pone.0210120 PMC6312313 30596786

[23] Zhang HL, Li WS, Xu DN, Zheng WW, Liu Y, Chen J, et al. Mucosa-repairing and microbiota-balancing therapeutic effect of Bacillus subtilis alleviates dextrate sulfate sodium-induced ulcerative colitis in mice. Exp Ther Med. 2016;12:2554–62. 10.3892/etm.2016.3686 PMC5038491 27698758

[24] Dhongade AR, Joshi SS, Kulkarni SV, Jadhav SS, Nargundkar N, Deshmukh D, et al. The Efficacy and Safety of a Bacillus Probiotic Combination for the Treatment of Acute Diarrhea in Children: A Double-blind, Randomized, Placebo-controlled Multicentric Study. Pediatr Infect Dis. 2022;4:86–91.

[25] Garvey SM, Mah E, Blonquist TM, Kaden VN, Spears JL. The probiotic Bacillus subtilis BS50 decreases gastrointestinal symptoms in healthy adults: a randomized, double-blind, placebo-controlled trial. Gut Microbes. 2022;14:2122668. 10.1080/19490976.2022.2122668 PMC9590435 36269141

[26] Lei K, Li YL, Wang Y, Wen J, Wu HZ, Yu DY, et al. Effect of dietary supplementation of Bacillus subtilis B10 on biochemical and molecular parameters in the serum and liver of high-fat diet-induced obese mice. J Zhejiang Univ Sci B. 2015;16:487–95. 10.1631/jzus.B1400342 PMC4471600 26055910

[27] Penet C, Kramer R, Parker J, Guthrie N. A randomized, double-blind, placebo-controlled, parallel study evaluating the efficacy of Bacillus subtilis MB40 to reduce abdominal discomfort, gas, and bloating. Altern Ther Health Med. 2021;27:146–57. 31719214

[28] Zhou Z, Pan X, Li L. Crosstalk between liver macrophages and gut microbiota: An important component of inflammation-associated liver diseases. Front Cell Dev Biol. 2022;10:1070208. 10.3389/fcell.2022.1070208 PMC9723159 36483677

[29] Celaj S, Gleeson MW, Deng J, O’Toole GA, Hampton TH, Toft MF, et al. The microbiota regulates susceptibility to Fas-mediated acute hepatic injury. Lab Invest. 2014;94:938–49. 10.1038/labinvest.2014.93 PMC4152405 25068658

[30] Grander C, Adolph TE, Wieser V, Lowe P, Wrzosek L, Gyongyosi B, et al. Recovery of ethanol-induced Akkermansia muciniphila depletion ameliorates alcoholic liver disease. Gut. 2018;67:891–901. 10.1136/gutjnl-2016-313432 28550049

[31] Wang Z, Qian J, Lu X, Zhang P, Guo R, Lou H, et al. A single-cell transcriptomic atlas characterizes liver non-parenchymal cells in healthy and diseased mice. bioRxiv. 2021:2021.2007.2006.451396.

[32] Dunn WB, Broadhurst D, Begley P, Zelena E, Francis-McIntyre S, Anderson N, et al. Procedures for large-scale metabolic profiling of serum and plasma using gas chromatography and liquid chromatography coupled to mass spectrometry. Nat Protoc. 2011;6:1060–83. 10.1038/nprot.2011.335 21720319

