# Supplementary material for: Bacillus subtilis attenuates acetaminophen-induced liver injury and preserves gut–liver homeostasis in mice
Source: Cell Commun Signal. 2026 Aug 12;24:599. doi: 10.1186/s12964-026-03141-2 (PMC13746500; doi:10.1186/s12964-026-03141-2)
Supplement: Supplementary file 1 [file 12964_2026_3141_MOESM1_ESM.docx]

***Bacillus subtilis* attenuates acetaminophen-induced liver injury** **and preserves gut-liver homeostasis in mice**

**Qin Zhuo^1,2,†^, Kaiyi Fu^1,†^, Ziqi Liu^3,†^, Huilu Zhang^1,*^**

^1^ Department of Digestive Diseases, Huashan Hospital, Fudan University, Shanghai, China.

^2^ Human Phenome Institute, Fudan University, Shanghai, China.

^3^ Department of Hepatobiliary and Pancreatic Surgery, the First Affiliated Hospital, Zhejiang University School of Medicine, Hangzhou, China.

^†^ These authors contributed equally to this work.

* Correspondence: zhanghuilu003@163.com

**Correspondence to:**

**Huilu Zhang**

Address: Department of Digestive Diseases, Huashan Hospital, Fudan University, 12 Wulumuqi Middle Road, Shanghai, 200040, China.

Email: zhanghuilu003@163.com.

**Supplemental Figures 1–6**

**Supplemental Tables 1–4**

**Fig. S1** Therapeutic effects of *B. subtilis* in a post-treatment model of APAP-induced acute liver injury. (**A**) Schematic diagram of the experimental design. Mice were fasted for 10–12 h and intraperitoneally injected with APAP (300 mg/kg). One hour after APAP administration, mice received *B. subtilis* R179 (1 × 10⁹ CFU/day) or normal saline (NS) by intragastric administration for 2, 5, or 7 consecutive days. Serum, colon, and liver tissues were collected at the indicated endpoints. i.p., intraperitoneal injection; i.g., intragastric administration; NS, normal saline. (**B**, **D**, and **F**) Serum levels of ALT (left) and AST (right) at 48 h (**B**), day 5 (**D**), or day 7 (**F**) of post-treatment with *B. subtilis* or NS. (**C**, **E**, and **G**) Representative H&E staining of liver sections and quantification of hepatic necrotic areas after 2 days (**C**), 5 days (**E**), or 7 days (**G**) of post-treatment. Scale bars, 1000 μm. Data are presented as mean ± SEM. Statistical significance was assessed using unpaired two-tailed Student’s *t* test.

**Fig. S2** Flow cytometric identification of hepatic immune cell populations following *B. subtilis* treatment. (**A**) Flow cytometric gating strategy for identification of hepatic myeloid cell populations, including Ly-6G⁺ neutrophils, Ly-6C⁺ monocytes, monocyte-derived macrophages (MdMs), and TIM4⁺ Kupffer cells (KCs). (**B**) Flow cytometric gating strategy for identification of hepatic lymphoid cell populations, including NK cells, CD4⁺ T cells, CD8⁺ T cells, and Foxp3⁺ Tregs. (**C**) UMAP plots based on flow cytometry data showing major hepatic myeloid cell subsets in APAP+NS and APAP+R179 groups. (**D**) Relative proportions of Ly-6G⁺ neutrophils, CD11b⁺F4/80^int^ monocytes, and CD206⁺ macrophages in the indicated groups. (**E**) UMAP plots based on flow cytometry data showing major hepatic lymphoid cell subsets in APAP+NS and APAP+R179 groups. (**F**) Relative proportions of CD8⁺ T cells, CD4⁺ T cells, and Foxp3⁺ Tregs among hepatic CD45⁺ immune cells in the indicated groups. Data are presented as mean ± SEM. Statistical significance was assessed using unpaired two-tailed Student’s *t* test.

**Fig. S3** *B. subtilis* treatment is associated with enrichment of antioxidant- and Nrf2-related transcriptional programs. (**A**) Gene set enrichment analysis (GSEA) showing enrichment of the Hallmark Reactive Oxygen Species Pathway (left) and Downstream of Nrf2/ARE Pathway (right) in APAP+R179 compared with APAP+NS groups. The curves represent the running enrichment scores. Normalized enrichment score (NES), *P* value, and adjusted *P* value for each pathway are shown. (**B**) Relative mRNA expression of several genes in mouse liver between the indicated groups. Data are presented as mean ± SEM. Statistical significance was assessed using unpaired two-tailed Student’s *t* test.

**Fig. S4** Supplementary analyses of gut microbiota composition following *B. subtilis* treatment. (**A**) The observed amplicon sequence variants and α-diversity of the gut microbiota, including Shannon, Chao1, and Simpson indices, in the indicated groups. (**B**–**C**) Cecal microbial composition at the phylum level (**B**) and genus level (**C**) in the indicated groups (n = 5). (**D**) Relative abundance of the order *Bacillales* in the 16S dataset. (**E**) Relative fecal *B. subtilis* DNA abundance determined by species-specific quantitative PCR and normalized to total bacterial 16S rRNA gene abundance. Data are presented as mean ± SEM. Statistical significance was assessed using one-way ANOVA with Tukey’s multiple-comparisons test where applicable.

**Fig. S5** Predicted microbial functional shifts after *B. subtilis* treatment. (**A**–**B**) Predicted KEGG pathways generated by Tax4Fun2 at level 2 (**A**) and metabolism-related level 3 (**B**) between APAP+R179 and APAP+NS groups. Bars show the predicted mean relative representation of each pathway, and error-bar plots show between-group differences with 95% confidence intervals. Multiple-testing-adjusted *P* values are shown. (**C**) Sankey diagram displaying the increased or decreased 2^nd^-level KEGG pathways between APAP+R179 and APAP+NS groups.

**Fig. S6** Integrated microbiome-metabolome analysis highlights amino-acid-enriched signatures linked to specific taxa. (**A**) Partial least squares discriminant analysis (PLS-DA) score plot from the multivariate analysis of cecal metabolites in ESI^-^ mode. (**B**) Permutation analysis assessing the robustness of the PLS-DA model. (**C**) Pathway enrichment analysis of differential metabolite features between the APAP+R179 and APAP+NS groups. (blue, pathways from microorganism-host co-metabolism; green, pathways from microorganisms). **(D)** Heatmap showing Spearman correlations between representative microbial genera and differential metabolite features. Color intensity represents the Spearman correlation coefficient. *, *P* < 0.05; **, *P* < 0.01.

**Table S1.** Antibodies used for flow cytometry analysis

| Antigen | Clone | Fluorochrome | Brand |
| --- | --- | --- | --- |
| MHC-II (I-A/I-E) | M5/114.15.2 | FITC | BioLegend |
| Ly-6G | 1A8 | PE/Cyanine7 | BioLegend |
| CD45 | 30-F11 | APC | BioLegend |
| CD11b | M1/70 | Brilliant Violet 510 | BioLegend |
| Ly-6C | HK1.4 | Brilliant Violet 605 | BioLegend |
| F4/80 | BM8 | Brilliant Violet 650 | BioLegend |
| CD206 | C068C2 | PE | BioLegend |
| TIM4 | F31-5G3 | PE | BioLegend |
| CD8a | 53-6.7 | APC/Cyanine7 | BioLegend |
| CD4 | RM4-5 | Super Bright 600 | eBioscience |
| CD3 | 17A2 | FITC | BioLegend |
| Foxp3 | FJK-16s | APC | eBioscience |
| CD49b | HMα2 | PerCP/Cyanine5.5 | BioLegend |
| CD16/CD32 | 93 | Unconjugated | BioLegend |

**Table S2.** Primer pairs of real-time quantitative PCR analysis

| Gene name | Forward primer sequence (5′- 3′) | Reverse primer sequence (5′- 3′) |
| --- | --- | --- |
| *Actb* | GTGACGTTGACATCCGTAAAGA | GCCGGACTCATCGTACTCC |
| *Mafb* | TTCGACCTTCTCAAGTTCGACG | GAGATGGGTCTTCGGTTCAGT |
| *Ccr2* | ATCCACGGCATACTATCAACATC | TCGTAGTCATACGGTGTGGTG |
| *Spic* | AAACATTTCAAGACGCCATTGAC | AGTAGTTAGCGTTGCCTCTGA |
| *Nqo1* | AGGATGGGAGGTACTCGAATC | TGCTAGAGATGACTCGGAAGG |
| *Gclc* | CTACCACGCAGTCAAGGACC | CCTCCATTCAGTAACAACTGGAC |
| *Gclm* | AGGAGCTTCGGGACTGTATCC | GGAAACTCCCTGACTAAATCGG |
| *B. subtilis* | GGCCTATTGAACACCCTGATTTA | CGGATGCGGCCTTCTTTTTC |

**Table S3.** Results of the KEGG gene-list enrichment analysis

| KEGG term | *P*-value |
| --- | --- |
| Cytokine-cytokine receptor interaction | 0.00064 |
| Neomycin, kanamycin and gentamicin biosynthesis | 0.00078 |
| Galactose metabolism | 0.00131 |
| Starch and sucrose metabolism | 0.00142 |
| MAPK signaling pathway | 0.00278 |
| Hematopoietic cell lineage | 0.00335 |
| Type II diabetes mellitus | 0.00387 |
| Amino sugar and nucleotide sugar metabolism | 0.00409 |
| Basal cell carcinoma | 0.00795 |
| Maturity onset diabetes of the young | 0.01376 |
| Circadian rhythm | 0.01661 |
| Morphine addiction | 0.02113 |
| Neuroactive ligand-receptor interaction | 0.02305 |
| Metabolic pathways | 0.02559 |
| Nicotine addiction | 0.02771 |
| Viral protein interaction with cytokine and cytokine receptor | 0.02804 |
| Parathyroid hormone synthesis, secretion and action | 0.03082 |
| Carbohydrate digestion and absorption | 0.03148 |
| Glutamatergic synapse | 0.03601 |
| Alcoholism | 0.03744 |
| Cocaine addiction | 0.03818 |
| Cholesterol metabolism | 0.03958 |
| cAMP signaling pathway | 0.23084 |

**Table S4.** Results of the metabolite traceability analysis

| Name of the metabolite | Origin |
| --- | --- |
| N-Acetylmuramic acid | Microbiota |
| D-Malic acid | Microbiota |
| Hydrocinnamic acid | Microbiota |
| Pimelic acid | Microbiota |
| 4-Methylcatechol | Microbiota |
| L-Homoserine | Microbiota |
| p-Coumaric acid | Microbiota |
| 3-(2-Hydroxyethyl) indole | Microbiota |
| Indole | Microbiota |
| Thymidine | Co-Metabolism |
| L-Phenylalanine | Co-Metabolism |
| D-Glutamine | Co-Metabolism |
| Phenylpyruvic acid | Co-Metabolism |
| 8Z,11Z,14Z-Eicosatrienoic acid | Co-Metabolism |
| Orotic acid | Co-Metabolism |
| 2-Keto-4-methylthiobutyric acid | Co-Metabolism |
| Mevalonic acid | Co-Metabolism |
| 2-Deoxycytidine | Co-Metabolism |
| 4-Hydroxyphenylpyruvic acid | Co-Metabolism |
| L-Isoleucine | Co-Metabolism |
| L-Methionine | Co-Metabolism |
| L-Lysine | Co-Metabolism |
| L-Valine | Co-Metabolism |
| L-Pyroglutamic acid | Co-Metabolism |
| L-Tyrosine | Co-Metabolism |
| D-Serine | Co-Metabolism |
| Acetylcholine | Co-Metabolism |
| Pantothenic acid | Co-Metabolism |
| L-Histidine | Co-Metabolism |
| Histamine | Co-Metabolism |
| Dihydrosphingosine | Co-Metabolism |
| N-Acetylglutamic acid | Co-Metabolism |
| 4-Pyridoxic acid | Co-Metabolism |
| Anthranilic acid | Co-Metabolism |
| Uridine | Co-Metabolism |
| 1-Methylhistamine | Co-Metabolism |
| Riboflavin | Co-Metabolism |
